# An Alternative STAT Signaling Pathway Acts in Viral Immunity in *Caenorhabditis elegans*

**DOI:** 10.1128/mBio.00924-17

**Published:** 2017-09-05

**Authors:** Mélanie Tanguy, Louise Véron, Przemyslaw Stempor, Julie Ahringer, Peter Sarkies, Eric A. Miska

**Affiliations:** a Wellcome Trust/Cancer Research UK Gurdon Institute, University of Cambridge, Cambridge, United Kingdom; b Department of Genetics, University of Cambridge, Cambridge, United Kingdom; c École Normale Supérieure de Cachan, Université Paris-Saclay, Saclay, France; d MRC London Institute of Medical Sciences, London, United Kingdom; e Institute for Clinical Sciences, Imperial College London, United Kingdom; CIML

**Keywords:** *Caenorhabditis elegans*, Orsay virus, RNA virus, STAT signaling, innate immunity

## Abstract

Across metazoans, innate immunity is vital in defending organisms against viral infection. In mammals, antiviral innate immunity is orchestrated by interferon signaling, activating the STAT transcription factors downstream of the JAK kinases to induce expression of antiviral effector genes. In the nematode *Caenorhabditis elegans*, which lacks the interferon system, the major antiviral response so far described is RNA interference (RNAi), but whether additional gene expression responses are employed is not known. Here we show that, despite the absence of both interferon and JAK, the *C. elegans* STAT homolog STA-1 orchestrates antiviral immunity. Intriguingly, mutants lacking STA-1 are less permissive to antiviral infection. Using gene expression analysis and chromatin immunoprecipitation, we show that, in contrast to the mammalian pathway, STA-1 acts mostly as a transcriptional repressor. Thus, STA-1 might act to suppress a constitutive antiviral response in the absence of infection. Additionally, using a reverse genetic screen, we identify the kinase SID-3 as a new component of the response to infection, which, along with STA-1, participates in the transcriptional regulatory network of the immune response. Our work uncovers novel physiological roles for two factors in viral infection: a SID protein acting independently of RNAi and a STAT protein acting in *C. elegans* antiviral immunity. Together, these results illustrate the complex evolutionary trajectory displayed by innate immune signaling pathways across metazoan organisms.

## IMPORTANCE

Since innate immunity was discovered, a diversity of pathways has arisen as powerful first-line defense mechanisms to fight viral infection. RNA interference, reported mostly in invertebrates and plants, as well as the mammalian interferon response and JAK/STAT pathway are key in RNA virus innate immunity. We studied infection by the Orsay virus in *Caenorhabditis elegans*, where RNAi is known to be a potent antiviral defense. We show that, in addition to its RNAi pathway, *C. elegans* utilizes an alternative STAT pathway to control the levels of viral infection. We identify the transcription factor STA-1 and the kinase SID-3 as two components of this response. Our study defines *C. elegans* as a new example of the diversity of antiviral strategies.

## INTRODUCTION

RNA viruses, a highly diverse family known to infect organisms of almost all kingdoms of life, also represent an important burden on human health. Indeed, their highly mutagenic and adaptive nature is an ever-growing challenge for diagnosis and treatment and the underlying understanding of host-pathogen interaction. The first and critical step in mounting successful antiviral defense is the conserved innate immune response; however, its complexity is yet to be fully apprehended. Several pathways have been reported for various organisms that differ in their presence or relative importance. To date, the main pathways can be divided in two categories: protein-based innate immunity and RNA-based innate immunity. Jawed vertebrates rely mostly on the powerful interferon (IFN) system, whereas most other eukaryotes take advantage of the RNA interference (RNAi) machinery (1, 2).

In mammals, the initiation of the interferon response in response to RNA viruses depends on the RNA helicase RIG-I (retinoic acid-inducible gene I product) and its paralogs. RIG-I senses double-stranded RNA (dsRNA) intermediates that are generated during the replication of single-stranded RNA (ssRNA) viruses and initiates production of the type 1 interferon and inflammatory cytokines via activation of the transcription factors interferon regulatory factor IRF3/7 and NF-κB (3, 4). Interferons can in turn activate the JAK/STAT signaling pathway to induce an antiviral state and mediate viral control. In essence, binding of interferon to the type 1 interferon receptor (IFNAR) leads to activation of the receptor-associated tyrosine kinases JAK1 and TYK2, and eventually to the phosphorylation of the STAT transcription factors. Upon dimerization, STAT transcription factors can undergo translocation to the nucleus where they activate the expression of antiviral genes and inflammatory response genes (5). On the other hand, antiviral RNAi relies on the processing by the endoribonuclease Dicer of long double-stranded viral RNA molecules occurring during the viral cycle. These dsRNA molecules appear particularly during replication of the RNA genome by the virally encoded RNA-dependent RNA polymerase. After processing by Dicer, the resulting small interfering RNAs (siRNA) are loaded in the RNA-induced silencing complex (RISC) through the binding of the siRNA to a protein of the Argonaute family. Recognition of the target viral RNA by the active RISC eventually leads to its degradation (1).

In principle, all the components required for RNAi are still present in higher vertebrates, but this might simply reflect other biological functions such as microRNA (miRNA)-based gene regulation. It has also been proposed that the IFN-based innate immunity and antiviral RNAi might be incompatible (6, 7) or that the evolution of Dicer in mammalian somatic cells renders it inactive for long dsRNA processing (8).

However, some evidence supports potential roles for RNAi-based antiviral immunity in mammals (9–11). Similarly, the prominence of the RNAi pathway in fighting viruses in invertebrates may obscure other mechanisms that the innate immune system may use to combat viruses in these organisms. One such example is the nematode *Caenorhabditis elegans*, where antiviral immunity has previously been shown to involve a potent RNAi response (12).

**FIG 1:**
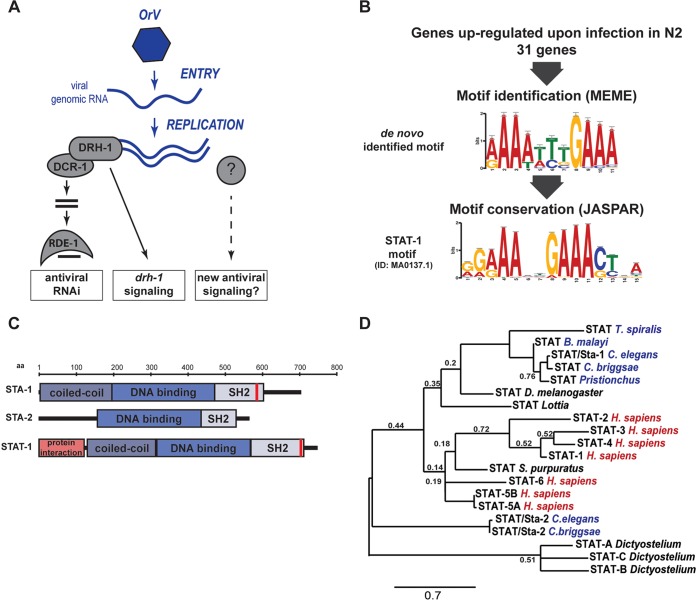
Identification of a STAT transcription factor signature in immune response against the Orsay virus (OrV) in *C. elegans*. (A) Schematic representation of known antiviral pathways in *C. elegans*. (B) MEME motif enrichment in genes regulated upon infection and conservation of the *de novo*-identified motif by Tom-tom. ID, identifier. (C) Schematic representation of conserved domains between the human STAT-1 and *C. elegans* STATs. The red bars depict the predicted phosphorylation sites (20). aa, amino acids. (D) Phylogenetic analysis of STAT transcription factors. Nematodes are indicated in blue, and vertebrates are indicated in red. *Trichinella spiralis*, *Brugia malayi*, *Caenorhabditis briggsae*, *Drosophila melanogaster*, *Homo sapiens*, and* Strongylocentrotus purpuratus* STATs are shown on the tree. The tree is based on full-length sequences. Alignment was performed with Muscle. Branch support values are from bootstrap using 1,000 iterations. Only values lower than 0.8 are depicted. The bar shows a branch length of 0.7 nucleotide substitutions per position.

A single virus, the Orsay virus, has been so far described to infect *C. elegans* in the wild (12). This small bipartite single-stranded RNA virus is efficiently targeted by the nematode RNAi machinery to prevent its replication. Surprisingly, DRH-1, a conserved helicase related to RIG-I, is essential for the antiviral RNAi pathway in *C. elegans* instead of the classical interferon response triggered by its mammalian counterpart RIG-I in mammals (Fig. 1A) (13–15). Interestingly, a previous analysis of gene expression changes upon viral infection revealed evidence for the induction of antiviral response genes upon viral infection independent of the RNAi pathway (16). However, neither the upstream signaling pathway linking these gene expression changes to viral infection nor the extent to which gene expression alterations directly contribute to antiviral defense are known. We therefore set out to uncover new signaling pathways involved in sensing and transducing viral infection.

## RESULTS

### Identification of a STAT transcription factor as a modulator of the infection response.

In order to identify key factors regulating the immune state of *C. elegans* after infection by Orsay virus (OrV), we set out to identify conserved regulatory motifs for the genes that participate in the transcriptional response to infection. Previously we identified a set of such putative antiviral response genes that were upregulated upon infection with the Orsay virus in the laboratory reference strain N2 (16). Interestingly, some of these genes, also upregulated upon infection of the antiviral RNAi-deficient *rde-1* mutant, were not upregulated in another domesticated strain of *C. elegans*, JU1580, which is also hypersensitive to the Orsay virus (13, 16). The JU1580 strain lacks the RIG-I homolog DRH-1 (Fig. 1A). This suggested that DRH-1 might be required for a transcriptional response to infection, independently of RNAi. However, as strains JU1580 and N2 differ at many loci besides *drh-1*, including some infection response genes (12, 13), we decided to test this further by performing microarray analysis to compare infection-induced genes in strain N2, a *drh-1* knockout in the N2 strain, JU1580, and JU1580 carrying a transgene containing the N2 *drh-1* locus (JU1580 *drh-1* rescue strain). *drh-1* mutants infected with Orsay virus showed changes in gene expression similar to those of infected JU1580 animals (see Fig. S1 in the supplemental material), in particular, the lack of upregulation of a set of genes induced in wild-type animals. Importantly, this shows that there must be gene expression changes that are induced independently of the level of viral replication, as both JU1580 and *drh-1* have much higher virus titers than strain N2 or the JU1580 *drh-1* rescue strain. This indicates that there may be signaling pathways governing antiviral gene expression in *C. elegans* (Fig. S1).

10.1128/mBio.00924-17.1 FIG S1  Orsay virus (OrV) infection induces gene expression changes that depend on the genotype of the host. The heatmap shows changes in gene expression, as measured by Affymetrix array, occurring in each of the indicated strains after infection by the Orsay virus. Download FIG S1, TIF file, 1 MB. Copyright © 2017 Tanguy et al. 2017 Tanguy et al. This content is distributed under the terms of the Creative Commons Attribution 4.0 International license.

To gain insights into the nature of the signaling events following viral infection, we extracted the promoters from the set of genes upregulated in strain N2 after OrV infection (Data Set S1) and searched for associated motifs (E value of <0.1 against a background model drawn from the nucleotide composition of the input sequences) using the motif prediction software MEME (17). We then compared these motifs to known transcription factor DNA binding motifs in the JASPAR core database using Tom-tom (18, 19). Remarkably, we identified an enriched motif with similarity to the one of STAT transcription factors (Fig. 1B), well-known to have a conserved role in antiviral defense in mammals. There are two STAT homologs, STA-1 and STA-2, in *C. elegans* (Fig. 1C). STA-1 has all the classical functional STAT domains: a coiled-coil domain for protein-protein interaction, a DNA binding domain, an SH2 domain, and a putative tyrosine phosphorylation motif (20). STA-2 is similar but lacks the coiled-coil domain as well as the tyrosine phosphorylation motif (21). Consistent with this, a phylogenetic tree with *C. elegans* STA-1 and STA-2 and representative STATs from other organisms suggests that STA-1 is closely related to mammalian STATs but that STA-2 is more divergent (Fig. 1D). Interestingly, STA-2 has previously been implicated in an antifungal response (21), whereas STA-1 has been linked to developmental signaling in *C. elegans* (22). We therefore speculated that STA-1 and/or STA-2 might have a previously unappreciated role in antiviral defense.

10.1128/mBio.00924-17.7 DATA SET S1  Upstream sequences of genes upregulated upon infection Download DATA SET S1, TXT file, 0.03 MB. Copyright © 2017 Tanguy et al. 2017 Tanguy et al. This content is distributed under the terms of the Creative Commons Attribution 4.0 International license.

**FIG 2:**
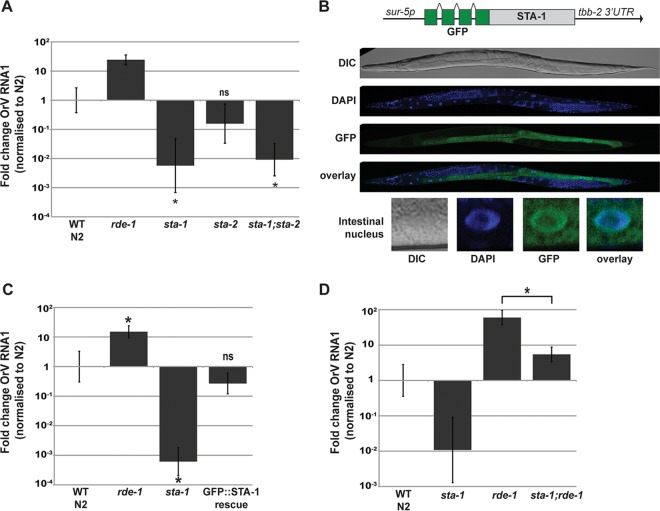
STA-1 is a key transcription factor in the immune response upon Orsay virus (OrV) infection. (A, C, and D) Viral loads in different strains measured by RT-qPCR on the Orsay virus RNA1 genome after 3 days of infection. The wild-type (WT) control N2 strain, *rde-1*(*ne219*) strain, and strains with *sta-1*(*ok587*) and *sta-2*(*ok1860*) mutations were infected. Values that were significantly different (*P* < 0.01) from the value for the wild-type control strain (unless otherwise indicated) by a two-tailed Mann-Whitney U test are indicated with an asterisk. Values that were not significantly different (ns) are indicated. *n* = 6 for panels A and C, and *n* = 4 for panel D. (B) Expression of a single-copy transgene coding for a GFP::STA-1 fusion protein under the control of a *sur-5* promoter in adult animals. DIC, differential interference contrast.

To evaluate a potential role for STAT signaling in antiviral defense in *C. elegans*, we used reverse transcription-quantitative PCR (RT-qPCR) to quantify the viral loads of *sta-1* and *sta-2* mutants following infection by the Orsay virus. For controls, we used wild-type N2 animals and *rde-1* mutants, which are hypersensitive to infection due to the lack of the Argonaute protein RDE-1, essential for the initiation of antiviral RNAi (Fig. 1A) (12, 23). Surprisingly, *sta-1* mutants were 100-fold less permissive to infection than wild-type animals, whereas* sta-2* mutants showed wild-type sensitivity (Fig. 2A). Double mutants lacking both STAT homologs were no more permissive than *sta-1* single mutants were. We conclude that STA-1 but not STA-2 acts in regulating antiviral defense. Consistent with these observations of viral load, induction of a viral response reporter gene, comprising *gfp* driven by the promoter of the viral response gene *sdz-6* (16), was reduced in *sta-1* mutants compared to strain N2 (*P* = 0.0061) and increased in *rde-1* mutants (*P* = 0.0034) (Fig. S2). Next we generated a single-copy, intrachromosomal *sur-5*::*gfp*::*sta-1* transgene, which drives expression of a green fluorescent protein (GFP)–STA-1 fusion protein in the intestine and other somatic tissues (Fig. 2B). GFP–STA-1 accumulated on chromatin in the nuclei of intestinal cells (Fig. 2B). Importantly, the *sur-5*::*gfp*::*sta-1* transgene restored wild-type sensitivity to the Orsay virus in a strain lacking endogenous *sta-1* (Fig. 2C). Finally, we asked whether STA-1 acts independently of the antiviral RNAi pathway using epistasis analysis (Fig. 2D). In *rde-1* mutants lacking the antiviral RNAi response (Fig. 1A), the Orsay virus accumulates to much higher levels than in the wild-type N2 strain. However, *sta-1; rde-1* double loss-of-function mutants show significantly reduced viral loads compared to *rde-1* single mutants, suggesting that *sta-1* acts in parallel or downstream of *rde-1* (Fig. 2D). Together, these results identify the *C. elegans* STAT transcription factor STA-1 as acting in addition to the antiviral RNAi pathway in the immune response to viral infection in *C. elegans*.

10.1128/mBio.00924-17.2 FIG S2  *sta-1* reduces activation of an infection sensor. Activation of the *sdz-6* viral sensor. The promoter of the *sdz-6* gene drives the expression of the GFP in the infected intestine. Animals were scored for GFP signal after 3 days of infection in three biological replicates with an average of 125 animals scored for each condition. A *t* test was performed to compare the average fraction of positive animals (on and dim) after infection in wild-type (WT) and *rde-1* animals and in WT and *sta-1* animals. Download FIG S2, TIF file, 0.8 MB. Copyright © 2017 Tanguy et al. 2017 Tanguy et al. This content is distributed under the terms of the Creative Commons Attribution 4.0 International license.

### STA-1 is a repressor of infection response genes.

**FIG 3:**
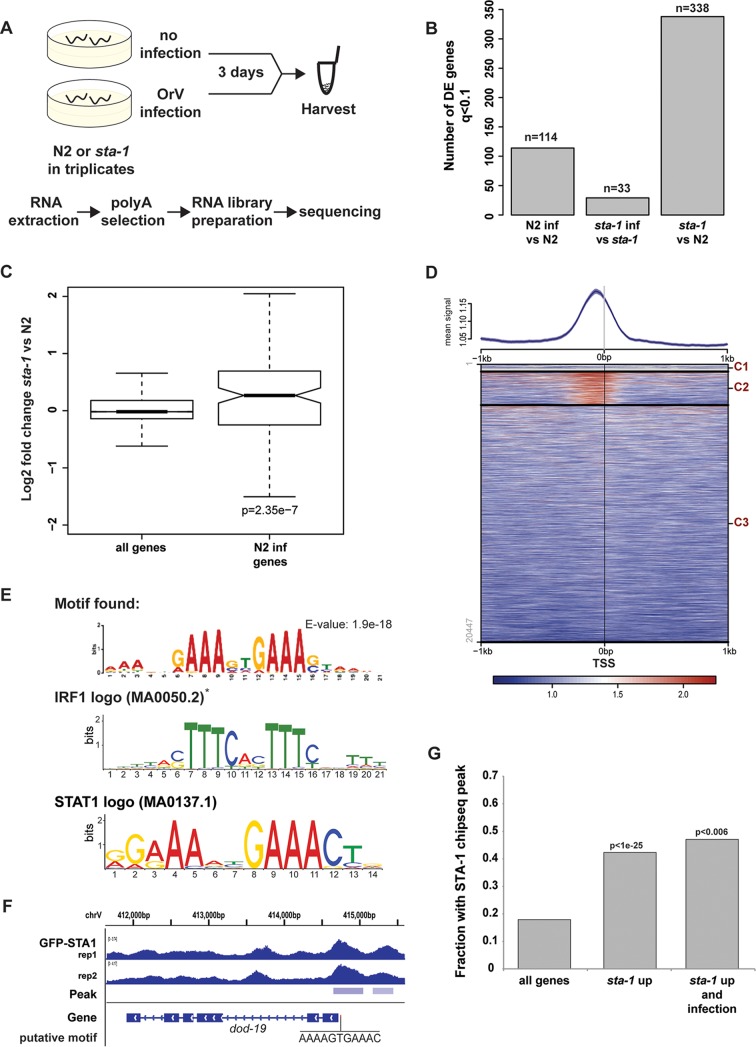
STA-1 acts at the promoter of virus response genes and represses their expression. (A) Schematic representation of the RNA-seq experiment performed with strain N2 and *sta-1*(*ok587*) animals. (B) Representation of the number of genes showing differential expression (DE) upon infection in strain N2 (N2 inf), infection in *sta-1* mutant animals, or infection in* sta-1* mutants compared to N2, as measured by RNA-seq. (C) Gene expression measured by RNA-seq in a *sta-1* mutant animals normalized to the N2 control. All genes or a subset of genes that show upregulation upon infection in N2 animals are depicted. The box shows the interquartile range, and the whiskers extend to the greatest value ≤1.5 times the interquartile range from the box. The significance of the enrichment is represented as the *P* value of the Wilcoxon unpaired test. (D) ChIP-sequencing after immunoprecipitation of the GFP::STA-1 transgene. (Top) The ChIP signal is plotted 1 kb upstream and 1 kb downstream of all genes anchored at TSS as in references 52 and 53. The heatmap was generated using k-means clustering (three clusters, C1 [cluster 1], C2, and C3]) of all transcription start sites (TSS) using the GFP::STA-1 signal for clustering. (E) Motif identification and conservation by Centrimo (54) for the 1-kb sequence centered around the summits of the peaks in cluster 2 (C2) in panel D. The asterisk indicates that the IRF1 motif identified by Tom-tom matches the reverse complement of the motif found. (F) Example of a upregulated *sta-1* gene showing binding of STA-1 by ChIP-seq in two independent biological replicates (rep1 [replicate 1 ] and rep2). The putative STA-1 binding motif is indicated. (G) ChIP peak enrichment in different sets of genes. The three sets of genes were genome-wide genes (all genes), genes upregulated in *sta-1* versus strain N2 (*sta-1* up), and genes differentially expressed upon infection and upregulated in *sta-1* (*sta-1* up and infection).

In order to test whether the low permissivity of *sta-1* mutants to viral infection reflects a role for STA-1 in the regulation of antiviral response genes, we performed transcriptome sequencing (RNA-seq) analysis of wild-type strain N2 and *sta-1* mutant animals with or without OrV infection (Fig. 3A). We then selected transcripts altered significantly after OrV infection (DESeq *q* value [false-discovery rate] of <0.1; Data Set S2). We observed a robust response to infection in N2 animals, whereas fewer transcripts were significantly altered in *sta-1* mutants upon infection (Fig. 3B). However, *sta-1* mutants displayed a constitutive deregulation of gene expression compared to N2 animals in the absence of OrV infection (Fig. 3B). Furthermore, genes that changed expression significantly upon infection in N2 showed a strong trend to be constitutively upregulated in *sta-1* mutants, while this was not the case when all genes were considered (Fig. 3C). These data suggest that STA-1 largely acts as a transcriptional repressor of an antiviral gene expression program. However, this antiviral gene expression program includes genes that are up- and downregulated upon infection, either directly or indirectly (Fig. 3B). We therefore propose that the lower permissivity of *sta-1* mutants to viral infection is caused by either a constitutive deregulation of the antiviral defense gene expression program or control by STA-1 of OrV essential host genes.

10.1128/mBio.00924-17.8 DATA SET S2  Differential gene expression measured by RNA-seq. Filtered data set for genes showing differential expression (*P* < 0.1) in at least one sample. Download DATA SET S2, XLSX file, 0.2 MB. Copyright © 2017 Tanguy et al. 2017 Tanguy et al. This content is distributed under the terms of the Creative Commons Attribution 4.0 International license.

Next we asked whether STA-1, similarly to STATs in mammals, might bind to DNA in a sequence-specific manner to regulate infection response genes. We therefore performed chromatin immunoprecipitation-DNA sequencing (ChIP-seq) analysis of GFP::STA-1 to determine its genomic binding pattern in noninfected animals expressing the *sur-5*::*gfp*::*sta-1* transgene but lacking endogenous *sta-1*. Through GFP::STA-1 ChIP-seq, we identified 2,133 STA-1 peaks across the genome. STA-1 was enriched close to transcription start sites (TSS) of about 20% of all genes, with an enriched binding peak located ~200 bp upstream of their TSS (Fig. 3D and Data Set S3). These data are in agreement with STA-1 acting as a specific DNA-binding transcription factor to regulate gene expression. To test further the association of STA-1 binding with the infection gene expression response, we used the sequences surrounding the STA-1 peaks in order to search for enriched motifs. Importantly, we recovered an enriched motif that was nearly identical to the motif predicted from our gene expression analysis (Fig. 1B) and matching the mammalian IRF and STAT motifs in the JASPAR core database and the consensus core interferon-sensitive response element (ISRE) TTCNNTTT (Fig. 3E and F) (24). Intriguingly, we additionally identified a separate highly enriched motif with strong similarity to the consensus sequence for GATA-like transcription factors (Fig. S3). The predicted GATA and STAT motifs were not found at the same set of genes, suggesting that GATA-like transcription factors may be able to recruit STA-1 to DNA independently of STA-1 DNA binding, consistent with previous studies of mammalian cells (25). This may serve to expand the number of sites bound by STA-1 beyond those containing a core STAT binding element.

10.1128/mBio.00924-17.3 FIG S3  Discovery of a GATA motif enriched in the STA-1-bound genes. (A) Enrichment of the IRF motif (as depicted in Fig. 3) across 1,375 peaks (out of 3,211 total peaks). (B) Enrichment of a GATA motif across 503 peaks (out of a total of 3,211 peaks) and logo representation. Download FIG S3, TIF file, 0.9 MB. Copyright © 2017 Tanguy et al. 2017 Tanguy et al. This content is distributed under the terms of the Creative Commons Attribution 4.0 International license.

10.1128/mBio.00924-17.9 DATA SET S3  Association between ChIP peak calling and gene names Download DATA SET S3, XLSX file, 0.5 MB. Copyright © 2017 Tanguy et al. 2017 Tanguy et al. This content is distributed under the terms of the Creative Commons Attribution 4.0 International license.

We next tested the association between STA-1 DNA binding and gene expression. STA-1 binding was strongly enriched at genes with increased expression in *sta-1* mutant animals (*P* < 1e−25 [Fig. 3G]) (Fig. S4) compared to all genes. However, it is worth noting that STA-1 binding was also enriched at the promoters of the smaller number of genes downregulated in the mutant (11/46 downregulated genes versus 122/379 upregulated genes). Thus, although STA-1 largely acts as a constitutive repressor of gene expression, it may have a more complex role at the promoters of some genes. The intersection of STA-1 binding with all N2 infection response genes, including those not upregulated in *sta-1* mutants was not significant, which may indicate that other signaling pathways are involved in gene expression responses to infection or technical limitations of identifying STA-1 binding sites from ectopically expressed GFP–STA-1; however, STA-1 binding was strongly enriched at N2 response genes that were also upregulated in *sta-1* mutants, although there were few of these genes (Fig. 3G).

10.1128/mBio.00924-17.4 FIG S4  STA-1 is bound to genes upregulated in *sta-1* mutants and infection genes. The table shows the intersection for gene sets and STA-1 ChIP-seq peaks as depicted in Fig. 3G. Download FIG S4, TIF file, 0.6 MB. Copyright © 2017 Tanguy et al. 2017 Tanguy et al. This content is distributed under the terms of the Creative Commons Attribution 4.0 International license.

### STA-1 is required for normal life span.

**FIG 4:**
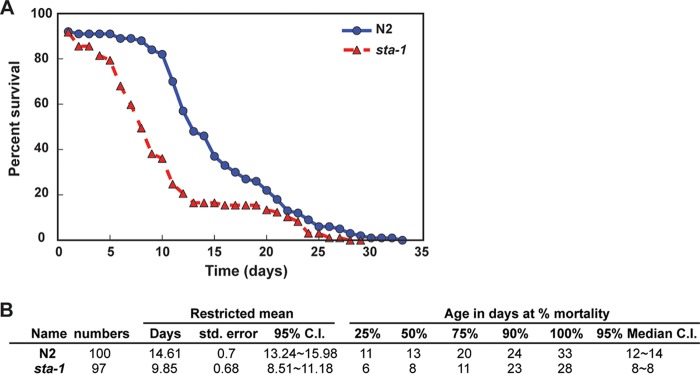
STA-1 is required for normal life span. (A) Survival plot showing the relationship between the survival and age of the wild-type N2 and *sta-1* mutant animals. (B) Descriptive statistics of the life span experimental data in panel A. The associated log rank test Bonferroni *P* value is 2.7e−6. std. error, standard error; 95% C.I., 95% confidence interval.

The lower permissivity of *sta-1* mutants to infection raises the question of whether there are negative fitness consequences associated with STA-1 deficiency that might act as trade-offs between resistance to infection and optimal growth. No obvious defects in development or fecundity were observed in *sta-1* mutants, consistent with published data (20). However, we observed a significant decrease in the median life span of *sta-1* mutants (Fig. 4). This may be due to the constitutive activation of pathogen response genes in *sta-1* mutants imposing a cost on animal development and/or physiology as has been shown in other systems, e.g., insects (26–28).

### The SID-3 kinase acts upstream of STA-1 in regulation of the accumulation of the Orsay virus.

**FIG 5:**
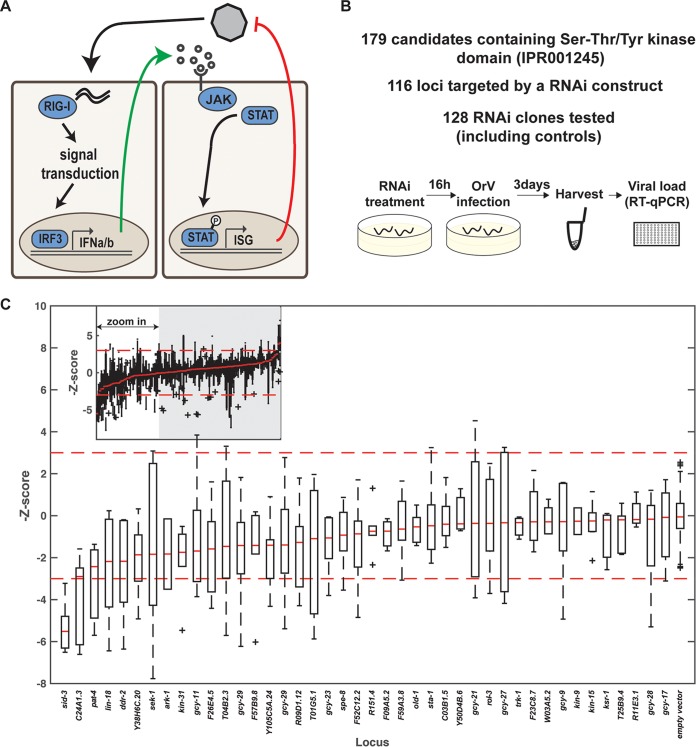
The tyrosine kinase SID-3 enables efficient viral replication. (A) Activation of the JAK/STAT signaling pathway in RNA virus infection in humans. Upon recognition of the viral RNA by a sensor like RIG-I, type 1 interferon (alpha/beta interferon) is released in the environment, recognized by the IFN receptor, and activates the JAK tyrosine kinase to trigger nuclear translocation of activated STAT transcription factors. IFNa/b, alpha/beta interferon; ISG, interferon-stimulated genes; P, phosphate. (B) Overview of the RNAi screen. (C) RNAi treatment leading to increased resistance to OrV infection. The viral RNA accumulation after RNAi treatment is represented by the Z-score of the ΔΔ*C*_*T*_ values (relative to empty vector [eV]). The red broken lines represent the 99% confidence interval of the control RNAi (eV), calculated as plus or minus 2.7 standard deviations. The red bars represent the median of the biological replicates for the genes tested or for controls. The plus signs represent outliers. The inset at the top of the graph depicts all tested clones.

**FIG 6:**
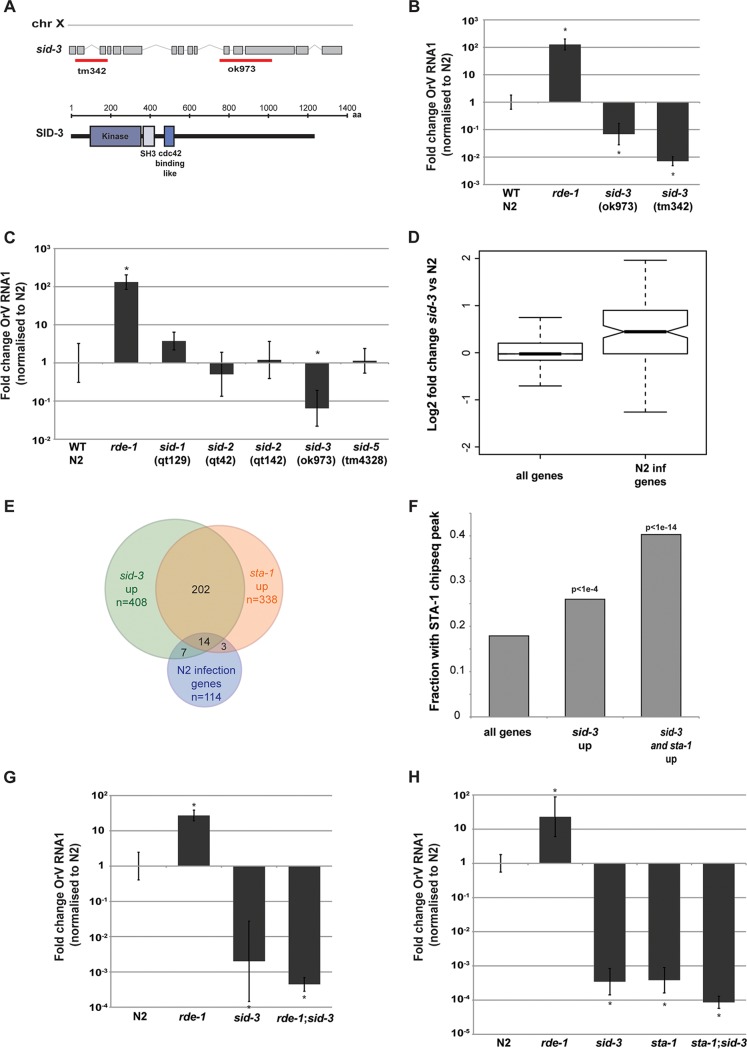
SID-3 and STA-1 regulate a common set of genes. (A) Schematic representation of the different *sid-3* alleles available. The *sid-3 ok973* allele induces a deletion of 1,330 bp and insertion of 12 nucleotides, deleting exons 11 and 12 and part of exon 13, after the tyrosine kinase domain. The *sid-3 tm342* allele is a null allele, where an early 821-bp-long deletion (exon 1 to exon 3) and insertion of 7 nucleotides lead to a premature stop codon. chr X, chromosome X. (B and C) Viral load after 3 days of infection, measured by RT-qPCR on the Orsay virus RNA1 genome. *, *P* < 0.01 by two-tailed Mann-Whitney test. *n* = 6 for each strain. (D) RNA-seq analysis showing an enrichment for infection genes in *sid-3*(*ok973*) upregulated genes. (E) Overlap between genes showing differential expression by RNA-seq upon infection or upregulated in *sta-1* or upregulated in *sid-3*. (F) Overlap between STA-1-bound genes, as shown by ChIP-seq and *sid-3* upregulated genes. (G and H) Viral load in the indicated strains, measured by RT-qPCR on the Orsay virus RNA1 genome after 3 days of infection. The *sid-3* allele used is *sid-3*(*ok973*). In panel G, *, *P* < 0.01 by two-tailed Mann-Whitney test and *n* = 6 for each strain. In panel H, *, *P* < 0.05 by two-tailed Mann-Whitney test and *n* = 3 for each strain except N2 and *n* = 4 for strain N2.

Having demonstrated that STA-1 acts as a constitutive repressor of antiviral response genes, we wondered how STA-1 activity was inhibited in response to viral infection. In mammals, STAT proteins are regulated by JAK tyrosine kinase phosphorylation (Fig. 5A). There is no conserved homolog of the JAK kinases in *C. elegans*; however, the canonical tyrosine phosphorylation site on STA-1 is conserved. Thus, other kinases may regulate STA-1 activity (20). There is also a growing body of evidence of JAK-independent phosphorylation of STAT transcription factors, including serine phosphorylation (29, 30). Additionally, tyrosine and serine/threonine kinases play an essential role in antibacterial and antifungal defense in *C. elegans* (31, 32). To identify potential kinases upstream of STA-1, we performed an RNAi screen, testing genes with the serine/threonine/tyrosine-protein kinase catalytic domain IPR001245 (33). The *C. elegans* genome encodes 176 proteins with this domain, 116 of which were available as clones as part of *C. elegans* genome-wide RNAi libraries (34, 35). Following RNAi by feeding, we infected animals with the Orsay virus and then quantified the viral load for each of the 116 candidates and additional controls (Fig. 5B and C and Data Set S4). Using a stringent cutoff (|Z-score| > 3), we identified only a single regulator of viral load, *sid-3*. RNAi of *sid-3* resulted in ~100-fold reduction of viral RNA accumulation compared to the control (Fig. 5C). We confirmed that *sid-3* is required for permissivity to viral infection by testing two independent deletion mutants of *sid-3* (Fig. 6A and B). SID-3 is a tyrosine kinase, implicated in systemic RNAi, and is presumed to assist in the import of dsRNA into the cell during experimental RNAi (36). However, this role in RNAi is not likely to be linked to its role in antiviral defense, because other genes required for systemic RNAi, such as *sid-1*, *sid-2*, and *sid-5*, do not show a significant difference in antiviral sensitivity from wild-type strain N2 (37) (Fig. 6C).

10.1128/mBio.00924-17.10 DATA SET S4  RNAi screen data Download DATA SET S4, XLSX file, 0.1 MB. Copyright © 2017 Tanguy et al. 2017 Tanguy et al. This content is distributed under the terms of the Creative Commons Attribution 4.0 International license.

We therefore wondered whether SID-3 might act in the same pathway as STA-1. We quantified gene expression in *sid-3* mutants using RNA-seq. Similar to what we observed for *sta-1* mutants, genes that changed expression significantly upon infection in strain N2 showed a strong trend to be constitutively upregulated in *sid-3* mutants, while this was not the case when all genes were considered (Fig. 6D and Data Set S2). Furthermore, the genes upregulated in *sid-3* mutant animals showed a striking overlap with the genes upregulated in *sta-1* mutant animals (*P* = 1.2e−15). Additionally, *sid-3* upregulated genes were enriched for antiviral response genes (*P* = 5.55e−10) (Fig. 6E and Fig. S5). Interestingly, among the viral infection response genes that showed constitutive upregulation in both *sta-1* and *sid-3* mutants (Data Set S2), three genes have been previously implicated in the control of adult life span (*C32H11.9*, *dod-21*, and *dod-23*), of which two are tandem paralogs of human EPXH1, an epoxide hydrolase gene, suggesting a putative role in detoxification. We also noticed the presence of *dct-17*, involved in the innate immune response and localized to the membrane raft, an important compartment for RNA virus replication (38, 39). Moreover, genes upregulated in *sid-3* mutants, including those shared with *sta-1*, were enriched for STA-1 binding by ChIP-seq, suggesting that *sid-3* acts upstream of* sta-1* in the antiviral gene expression response (Fig. 6F). We conclude that SID-3 and STA-1 act in the same process to regulate an innate antiviral immunity program.

**FIG 7:**
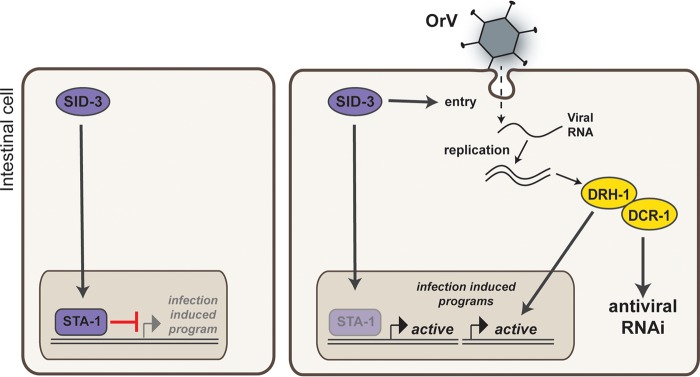
A STA-1 pathway controls the response to viral infection in *C. elegans*, a model.

Finally, we addressed the relationship between SID-3 and the antiviral RNAi pathway using epistasis analysis. Interestingly, *rde-1;sid-3* double loss-of-function mutants showed the same permissivity to OrV infection as *sid-3* mutants (Fig. 6G). This is in contrast to what we observed in *sta-1;rde-1* mutants (Fig. 2D). Additionally, *sta-1;sid-3* double mutants also showed a very low permissivity to OrV infection (Fig. 6H). One likely interpretation of these data is that SID-3 acts both upstream of antiviral RNAi and upstream of a STA-1-dependent antiviral gene expression program to regulate the response to OrV infection. We propose a model (Fig. 7) whereby in uninfected animals, SID-3 is required to maintain repression of viral infection response genes by STA-1. Upon infection, this signaling is curtailed, inducing an antiviral response program.

## DISCUSSION

Here, we uncover a hitherto unappreciated role for the *C. elegans* homolog of the mammalian STAT family of transcription factors. We demonstrate that the tyrosine kinase SID-3 and the STAT transcription factor STA-1 are two new key factors in the antiviral response in *C. elegans*. Additionally, we uncover a novel potential signaling pathway linking the kinase SID-3, previously reported only for systemic RNA interference, to STAT signaling. Our results have intriguing implications both for innate immunity in *C. elegans* and for the evolution of eukaryotic signaling pathways.

### Regulation of antiviral gene expression in *C. elegans.*

Previously there has been some debate over the extent to which gene expression responses to infection in *C. elegans* represent specific pathogen response pathways or more-general responses to stress. Our findings that a STAT family transcription factor is responsible for constitutively repressing infection response genes and that this is relieved upon infection suggest that at least some part of the transcriptional response to infection is specific. Moreover, constitutive upregulation of these genes, as demonstrated in mutants lacking *sta-1*, leads to low permissivity to viral infection, although further examination of the functions of these genes will be needed to understand exactly how their expression is involved in viral infectivity.

How is the constitutive repression of antiviral response genes by *sta-1* relieved upon viral infection? The fact that *sid-3* mutants show loss of *sta-1* repression suggests that phosphorylation of STA-1 is required for it to exert its repressive function. Whether the phosphorylation is directly mediated by a signaling cascade downstream of the kinase activity of SID-3 or conversely is caused by disruption of the SID-3 complex upon infection (as would potentially be mimicked by the *sid-3* mutation) is still a point of discussion. Viral infection may also lead to loss of SID-3 activity and concomitant loss of STA-1 phosphorylation. The differences in gene expression observed between *sta-1* mutant animals and infected animals could then be explained by the difference between the set of transcription factors active in an infected cell compared to the noninfected *sta-1* mutant.

An interesting clue to the potential role of SID-3 in viral infection comes from its proposed role in endocytosis (36). Nonenveloped viruses require the endosomal pathway for entry into cells; it is therefore plausible that viral entry into endosomes may lead to titration of SID-3 away from its role in signaling to STA-1, leading to relief of repression. Such an early role for *sid-3* in infection is consistent with the ability of *sid-3* mutation to suppress the sensitivity of RNAi pathway mutants. Indeed, Dave Wang and colleagues demonstrate in this issue that* sid-3* is required for viral entry (40). Thus, we hypothesize that the dependence of the virus on *sid-3* for its entry has been exploited by *C. elegans* as a mechanism to regulate antiviral gene expression. Further work will be required to refine this signaling pathway.

### Evolution of STAT signaling.

It is remarkable that the STAT family of transcription factors has a role in antiviral gene regulation in both mammals and *C. elegans*, despite the fact that both upstream and downstream genes in the pathway are not conserved in mammals and *C. elegans*. Even more remarkably, the mechanism whereby STAT transcription factors contribute to gene expression regulation are quite distinct in nematodes from that in mammals, as while STA-1 is constitutively bound to DNA and acts as a transcriptional repressor in *C. elegans*, STATs are activated in response to infection. Despite this, there appear to be intriguing parallels between STA-1 activity and the mammalian STAT pathway, in particular the association that we uncovered between STA-1 binding and GATA transcription and the conserved role of STATs in viral gene expression responses in *C. elegans* and mammals.

Our results highlight the remarkable plasticity of signaling pathways through evolution, whereby a central module may retain the same function over millions of years while acquiring distinct regulatory partners. This ability to both retain and diversify functionality simultaneously is clearly particularly important in the immune system, where the specific nature of the threats involved changes rapidly while their general modes of action may not. It will be intriguing to explore whether STATs in other metazoans have similarly diversified while retaining antiviral activity—it is possible that the *C. elegans* mode of STAT action that we uncover here is in fact the original conserved role of STAT transcription factors in viral responses. If so, vestiges of this function may be retained in mammalian cells, particularly those without expression of the JAK pathway.

## MATERIALS AND METHODS

### Nematode culture and strains.

We grew *C. elegans* under standard conditions at 20°C. The wild-type strain was var. Bristol N2. The food source used was *Escherichia coli* strain HB101 (Caenorhabditis Genetics Center, University of Minnesota, Twin Cities, MN, USA). Detailed information about all strains generated and used in this study can be found in Table S1 in the supplemental material.

10.1128/mBio.00924-17.6 TABLE S1  Strains used in this study Download TABLE S1, PDF file, 0.03 MB. Copyright © 2017 Tanguy et al. 2017 Tanguy et al. This content is distributed under the terms of the Creative Commons Attribution 4.0 International license.

### Viral filtrate.

Stably infected populations of sensitive animals (JU1580) were transferred to 2-liter liquid cultures with *E*. *coli* HB101 food and grown for 7 days. The supernatant of the culture was harvested on ice and filtered with a 0.22-µm filter. The resulting viral filtrate was aliquoted and stored at −80°C.

### Viral infection.

Two wild-type or three mutant larval stage 4 (L4) animals were added to seeded 50-mm plates. Sixteen hours later, 20 µl of Orsay virus filtrate was added to the edge of the bacterial lawn. Animals were collected 3 days after infection. The animals were washed off the plates with M9 buffer. The animal pellets were washed another three times by pelleting the animals either by gravity on ice or by centrifugation at 800 × *g* for 2 min in a swinging bucket centrifuge, snap-frozen in liquid nitrogen, and stored at −80°C.

### Life span assay.

One hundred animals of each strain were selected and transferred to ten 50-mm nematode growth medium (NGM) plates. Adult animals were transferred every 2 days to fresh plates for the duration of egg laying. Their survival was measured by movement of the head. Animals showing no head movement were gently touched twice with a worm pick and observed for 30 s following each touch. Animals that did not move were considered dead and removed from the plate. The survival curves were plotted and analyzed using OASIS (Online Application for the Survival Analysis) (41).

The experiment was reproduced three times with one representative example illustrated.

### RT-qPCR.

Lysis of the worm pellets was performed with 5 µl of worm pellet and 45 µl of the lysis solution from the Power SYBR green Cells-to-CT kit (Ambion). Ten freeze-thaw cycles and 30 min shaking at room temperature were performed before the lysis incubation step. The reverse transcription-quantitative PCR (RT-qPCR) was performed according to the manufacturer’s instructions. The qPCR was run on a Step One Plus real-time PCR system (Applied Biosystems). The analysis was done using the ΔΔ*C*_*T*_ method. The threshold cycle (*C*_*T*_) values of four to six biological replicates per experiment were pooled to generate the average Δ*C*_*T*_ for each strain and an associated standard error (SD). The ΔΔ*C*_*T*_ value was calculated with Δ*C*_*T*_ in strain N2 as the calibrator. The 2^−ΔΔ*CT*^ was plotted as the fold change, and the interval of confidence is represented by error bars as 2^−ΔΔCT + SD(Δ*CT*) ^and 2^−ΔΔ*CT* − SD(Δ*CT*)^. The significance of the differences observed was tested by a two-tailed Mann-Whitney U test at a significance level of *P* < 0.01. The experiments were repeated independently at least twice, and one representative example is shown in the figures.

### DNA constructs.

The viral sensor construct and the *gfp*::*sta-1* construct were generated by Gateway cloning using the MultiSite Gateway three-fragment vector construction kit (Life Technologies). Gateway entry clones containing each of the following were generated by standard techniques: *sur-5* promoter, *sdz-6* promoter, *sta-1* coding sequence, eGFP(F64L/S65T) (eGFP stands for enhanced green fluorescent protein), and *tbb-2* 3′ untranslated region (3′UTR). The single-copy transgene was generated by transposase-mediated integration (MosSCI), as described previously (42, 43), at insertion site ttTi5605 on chromosome II. Injection mixes contained 20 ng/µl of vector, 20 ng/µl of Mos1 transposase (only for MosSCI), and 5 ng/μl of a pharynx marker. Integration of the extrachromosomal array was performed by ethyl methanesulfonate (EMS) treatment (50 mM EMS for 4 h).

### Sensor scoring.

Scoring of the GFP was made by eye under a Leica MZ16F fluorescence stereomicroscope. Three biological replicates of the infection were analyzed, with at least 110 animals per replicate scored by eye, as follows: on for strong GFP signal, dim for weak/medium GFP signal, and off for no GFP.

### Imaging.

Adult animals were harvested from a plate; they were washed off in M9 buffer. After three additional washes in M9 buffer, the pelleted animals were fixed in 0.5 ml of precooled methanol at −20°C for 10 min. The pellet was washed three times in Tris-buffered saline containing 0.1% Tween 20 (TBST) and then incubated for 10 min in a 4′,6′-diamidino-2-phenylindole (DAPI) solution (0.5 mg/ml in TBST). The pellet of animals was washed three times in TBST. Five microliters of the pelleted animals was pipetted directly onto a Cel-line diagnostic microscope slide (Thermo Scientific) and imaged using a Leica SP8 upright microscope.

### Microarray.

To obtain a putative set of signaling response genes triggered by viral infection but not responding to the level of viral RNA, the array was first normalized to the expression of *cul-6*, as we and others have shown this to be closely linked to viral RNA levels (16, 44). Microarray data were processed using rma, and annotations were obtained through the Bioconductor pipeline in the R programming environment. Differentially regulated genes in any condition were identified by using *t* test, *P* < 0.01, and a twofold difference upon infection. Hierarchical clustering on the *drh-1* array was carried out on the set of differentially regulated genes using the hclust function in R and Ward’s method.

### Motif analysis.

To specifically identify potential viral response genes, we normalized the array by *cul-6*, as this removed variation in the final level of infection. Qualitatively, this identified trends similar to those previously published (16), such as putative antibacterial response genes upregulated specifically in strain JU1580 and *drh-1* upon infection. We identified genes that were >40% induced relative to *cul-6* in strain N2 upon infection and used BiomaRt to download the upstream sequences (Data Set S1). These sequences were used as input for MEME, attempting to find 0 or 1 site per gene. We screened these manually to avoid spurious hits and then compared potential motifs identified to the JASPAR core database. To test enrichment of STAT motifs in STA-1 chromatin immunoprecipitation-DNA sequencing (ChIP-seq) peaks, we used FIMO to search for the STAT-1 core motif within the 500-bp sequences upstream of genes containing a STA-1 binding site expressed at an increased level (DESeq *q* < 0.1) in *sta-1* mutants relative to strain N2, applying a false-discovery rate cutoff of 0.2. We compared this to a random set of genes chosen from the transcriptome sequencing (RNA-seq) data (see below).

### RNA-seq.

Animals were grown on 50-mm NGM plates, infected or not, and harvested as described in “Viral infection” above. Total RNA was extracted using TRIsure (Bioline, UK). RNA library preparation was performed with the NEBNext Ultra RNA Library Prep kit for Illumina with total RNA purified with the NEBNext poly(A) mRNA magnetic isolation module, according to the manufacturer’s instructions (NEB, USA).

### RNA-seq analysis.

RNA-seq data were aligned to the *C. elegans* transcriptome WS190 using bowtie2. Counts were obtained from resulting BAM files using BEDTools (45), and these were used to generate normalized data tables using DESeq (46) (Data Set S2). Significance between intersecting data sets was calculated by a Fisher’s exact test (Fig. S4 and Fig. S5). A cutoff of mean 25 normalized reads (normalized according to DESeq’s negative binomial distribution) for at least one condition was used, and significantly altered genes were selected (DESeq Benjamini-Hochberg multiple-test correction *q* < 0.1).

10.1128/mBio.00924-17.5 FIG S5  *sta-1*, *sid-3*, and infection by the Orsay virus lead to the regulation of a shared set of genes. (A) Table showing the percentage of intersection for gene sets as depicted in Fig. 6. (B) Table showing the intersection for gene sets and STA-1 ChIP-seq peaks as depicted in Fig. 6. The data used in Fig. 3 are shaded in the tables. Download FIG S5, TIF file, 0.9 MB. Copyright © 2017 Tanguy et al. 2017 Tanguy et al. This content is distributed under the terms of the Creative Commons Attribution 4.0 International license.

### ChIP sequencing.

Animals were grown on NGM plates seeded with thick *E*. *coli* HB101 food and harvested as a mixed-stage population. Frozen animals were ground to a fine powder, fixed in phosphate-buffered saline (PBS) containing 1% formaldehyde for 10 min, quenched with 0.125 µM glycine, and then washed three times in PBS with protease inhibitors. The pellet was resuspended in 1 ml of FA buffer (50 mM HEPES–KOH [pH 7.5], 1 mM EDTA, 1% Triton X-100, 0.1% sodium deoxycholate, 150 mM NaCl with protease inhibitors) per 4 ml of ground worm powder. Extract was sonicated to a size range of 200 to 1,000 bp using a Diagenode Bioruptor Pico with a setting of 18 pulses, each pulse lasting 30 s followed by a 30-s pause. The extract was spun for 10 min at 16,000 × *g* at 4°C, and the soluble fraction was flash frozen in liquid nitrogen and stored at −80°C until use. Each ChIP was prepared in 500 µl of FA buffer containing 1% Sarkosyl. Fifteen micrograms of an anti-GFP antibody (ab290) was incubated with 3 mg of extract. In addition, 10% of extract was saved as a reference. After overnight rotation at 4°C, 40 µl of blocked and washed magnetic protein A Dynabeads (Invitrogen) was added, and the incubation continued for 2 additional hours. The beads were washed at room temperature twice for 5 min in FA buffer, once in FA buffer with 500 mM NaCl for 10 min, once in FA buffer with 1 M NaCl for 5 min, once in TEL buffer (0.25 M LiCl, 1% NP-40, 1% sodium deoxycholate, 1 mM EDTA, 10 mM Tris-HCl [pH 8.0]) for 10 min, and twice in Tris-EDTA (TE) (pH 8.0) for 5 min. DNA was eluted twice with 57-μl elution buffer (1% SDS in TE with 250 mM NaCl) at 65°C for 15 min each time. Eluted DNA was incubated with 20 μg of RNase for 30 min at 37°C and then with 20 μg of proteinase K for 1 h at 55°C. Input DNA was also diluted in 114-µl elution buffer and treated with ChIP samples. Cross-links were reversed overnight at 65°C. DNA was purified on PureLink PCR purification columns (Invitrogen). The libraries were prepared using a modified TruSeq ChIP Library preparation kit protocol (https://ethanomics.files.wordpress.com/2012/09/chip_truseq.pdf). Size selection was performed using Agencourt AMPure XP beads (Beckman Coulter).

### ChIP-seq analysis. (i) Alignment to reference genome.

ChIP-seq and RNA-seq libraries were sequenced using Illumina HiSeq. Reads were aligned to the WS220/ce10 assembly of the *C. elegans* genome using BWA v. 0.7.7 (47) with default settings (BWA-backtrack algorithm). The SAMtools v. 0.1.19 “view” utility was used to convert the alignments to BAM format. Normalized ChIP-seq coverage tracks were generated using the R implementation of BEADS algorithm (48, 49). 

### (ii) Summed ChIP-seq input. 

We generated summed input BAM files by combining good-quality ChIP-seq input experiments from different extracts (eight experiments). The same summed inputs were used for BEADS normalization and peak calls. 

### (iii) Peak calls. 

Initial ChIP-seq peaks were called using MACS v. 2.1.1 (50) with permissive 0.7 *q*-value cutoff and fragment size of 150 bp against summed ChIP-seq input. To generate combined peak calls, we used the modified irreproducible discovery rate (IDR) procedure (https://www.encodeproject.org/software/idr/) with an IDR threshold of 0.05 to combine replicates (Data Set S3). The pipeline for generating IDR peaks is available here: https://github.com/Przemol/biokludge/blob/master/macs2_idr/macs2_idr.ipy. 

### (iv) Mean signal distribution plots and heatmaps.

The summarized signal profile and heatmap for GFP::STA-1 were generated using SeqPlots exploratory analyses and plotting software (51).

### RNAi screen.

RNAi clones from the Ahringer library (34, 35) were isolated on agar plates containing carbenicillin (50 μg/ml). Single colonies were picked in 2 ml LB plus ampicillin (50 μg/ml) and grown for 9 h with shaking at 37°C. Bacteria were then seeded onto 50-mm NGM plates containing isopropyl-β-d-thiogalactopyranoside (IPTG) (1 mM), carbenicillin (25 μg/ml), and amphotericin B (Fungizone) (2.5 μg/ml). Two days after seeding, two larval stage 4 (L4) animals were added and then grown at 20°C. After 16 h, plates were inoculated with 20 μl Orsay virus filtrate. Animals were collected after 3 days, and viral relative genome copy number was measured by reverse transcription-quantitative PCR (RT-qPCR). For each round of the screen, we used the following internal controls: N2 strain grown on empty vector (*E. coli* L4440 strain) as the normalization control, *drh-1* mutant fed on GFP RNAi as a positive control for infection, and N2 strain fed on *drh-1* RNAi clone as a positive control for RNA interference (RNAi). The full list of the RNAi clones and number of replicates used in this study is available in Data Set S4. Each replicate of the screen was performed with three biological replicates per RNAi clone treatment, and the screen was repeated at least twice. The plates with accidental fungal contamination and the plates where the RNAi treatment led to embryonic lethality were removed from the analysis. The resulting exact numbers of biological replicates are indicated in Data Set S4. Empty vector treatment was included on each qPCR plate analyzed and used as the calibrator. The ΔΔ*C*_*T*_ values were transformed into Z-scores, calculated as follows: Z-score_i _= (ΔΔ*C*_Ti _− µΔΔ*C*_Tempty vector, *n* = 161_)/standard deviation(ΔΔ*C*_Tempty vector_), where µ indicates mean and i is the gene. Boxplots of the Z-score for each treatment are represented in Fig. 5. Individual values used for the analysis are available in Data Set S4.

### Data availability.

High-throughput sequencing data sets are accessible through the GEO repository. All Illumina sequencing files are available from the GEO database (GSE95230 and GSE99586).
