# Supplementary figures and images for: An Alternative STAT Signaling Pathway Acts in Viral Immunity in Caenorhabditis elegans
Source: mBio. 2017 Sep 5;8(5):e00924-17. doi: 10.1128/mBio.00924-17 (PMC5587905; doi:10.1128/mBio.00924-17)

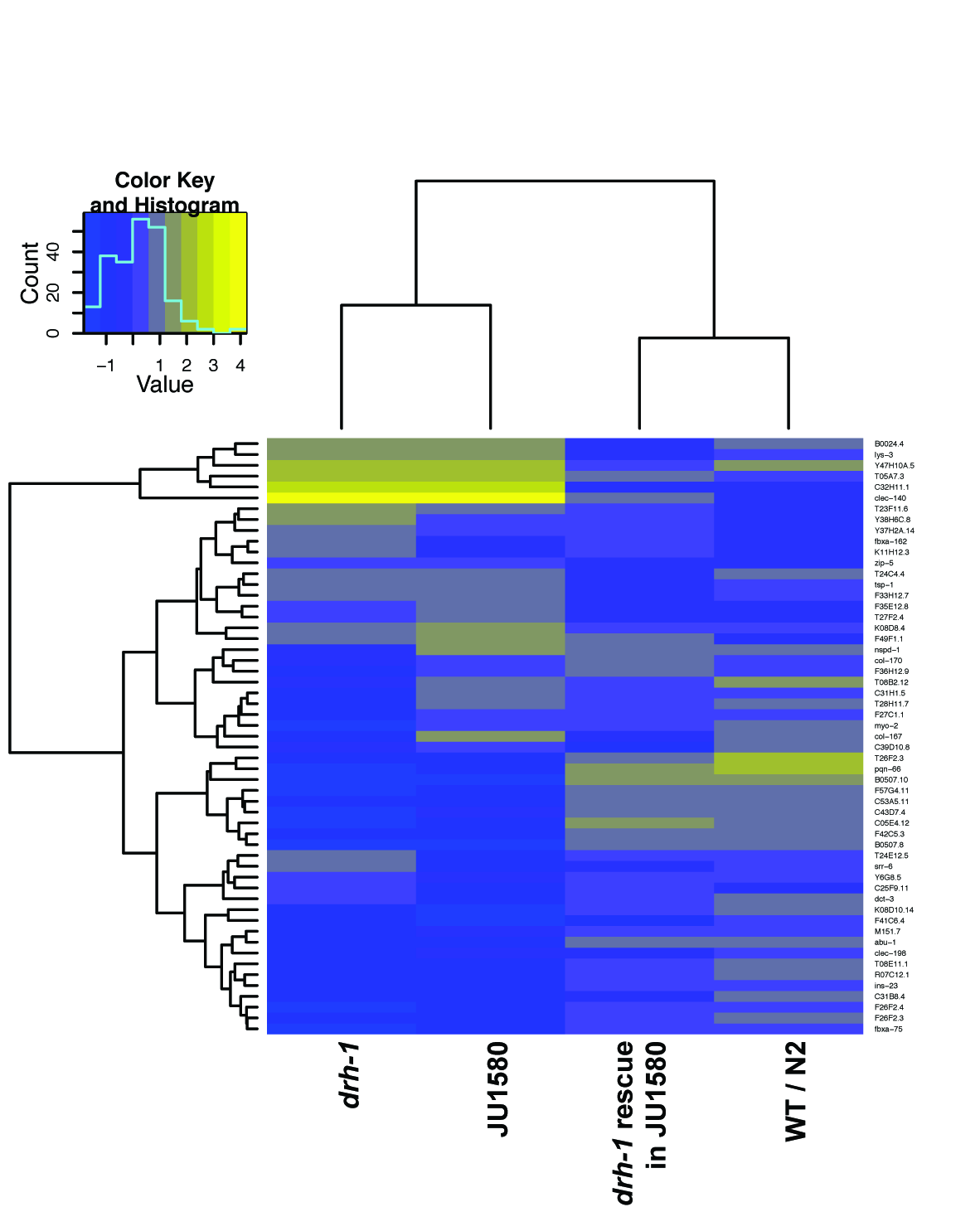

Supplement: FIG S1 [file mbo004173465sf1.tif]

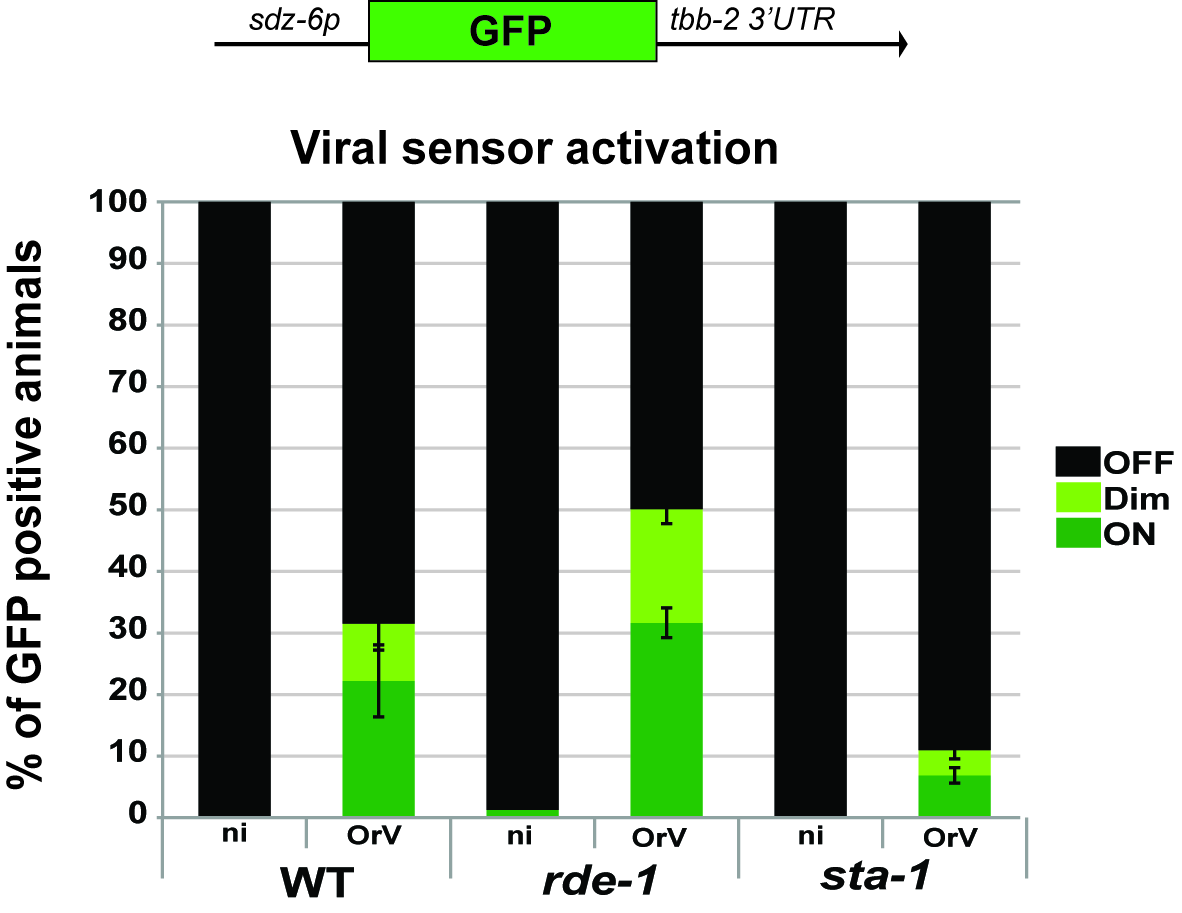

Supplement: FIG S2 [file mbo004173465sf2.tif]

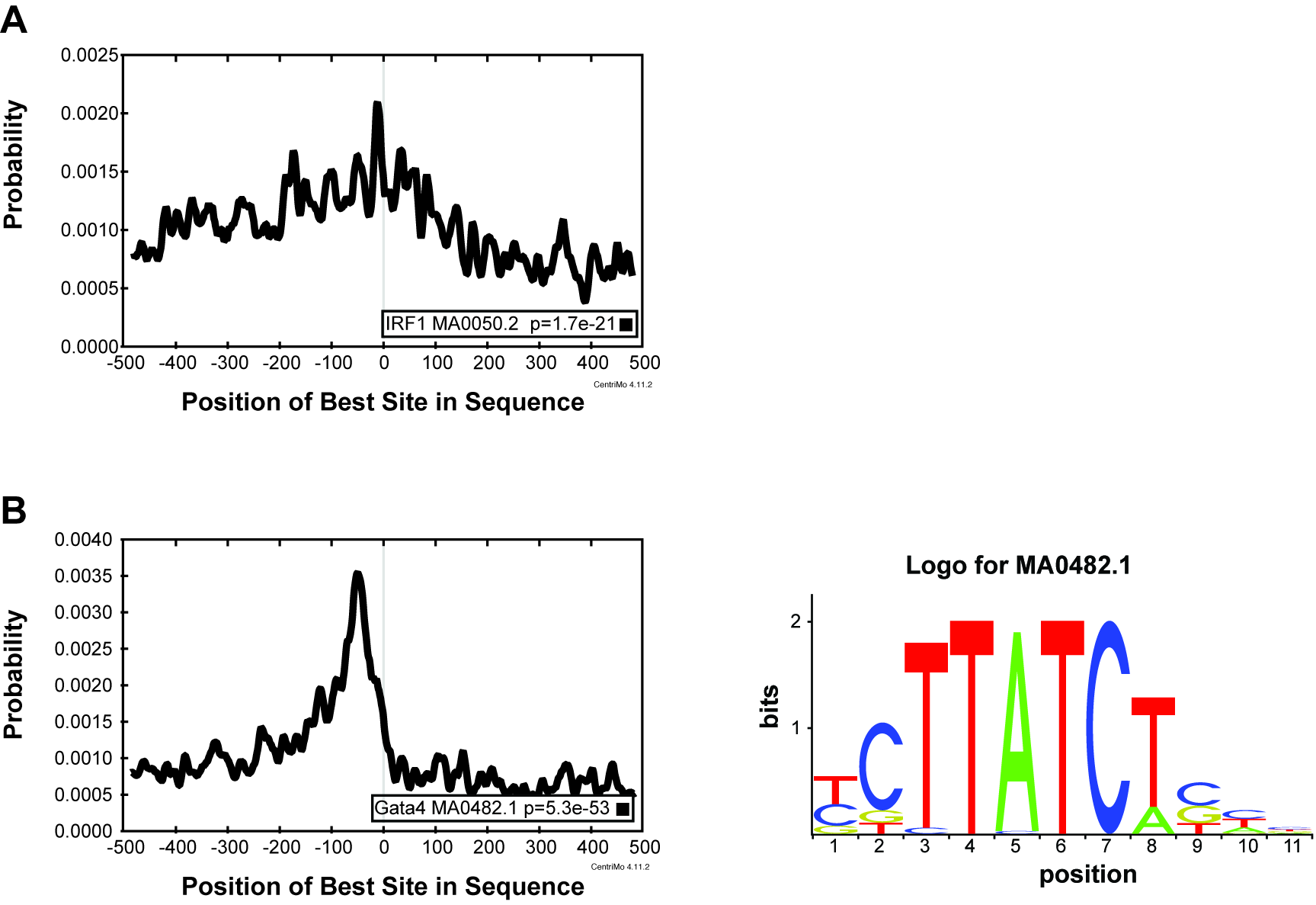

Supplement: FIG S3 [file mbo004173465sf3.tif]

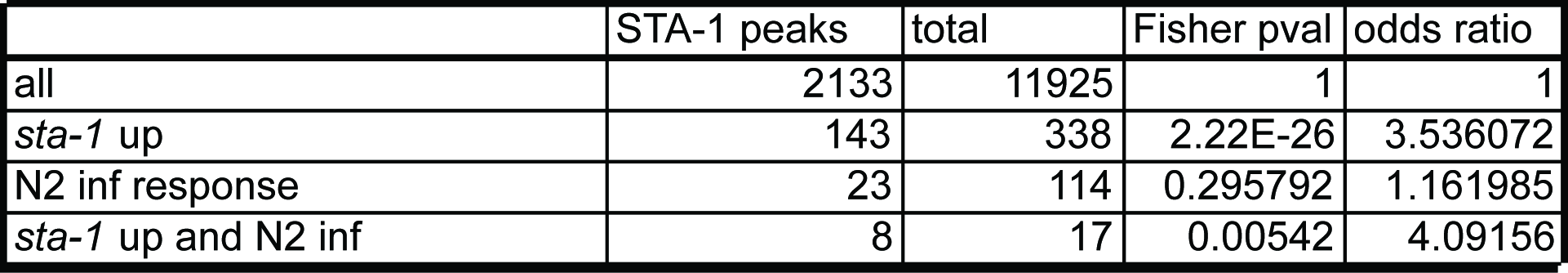

Supplement: FIG S4 [file mbo004173465sf4.tif]

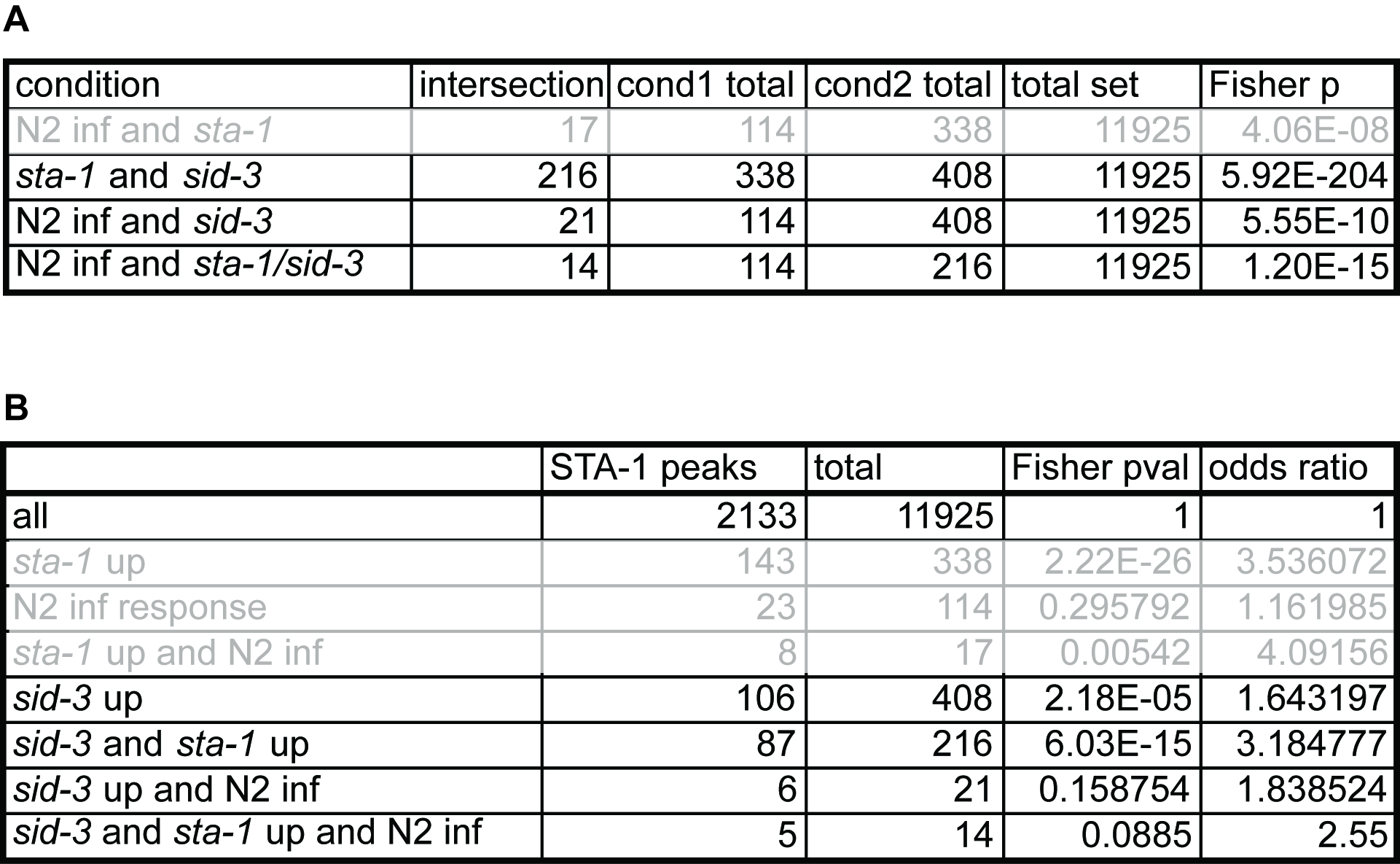

Supplement: FIG S5 [file mbo004173465sf5.tif]
