# Supplementary material for: An Alternative STAT Signaling Pathway Acts in Viral Immunity in Caenorhabditis elegans
Source: mBio. 2017 Sep 5;8(5):e00924-17. doi: 10.1128/mBio.00924-17 (PMC5587905; doi:10.1128/mBio.00924-17)
Supplement: TABLE S1 [file mbo004173465st1.pdf]

strain	genotype
EG6699	ttTi5605 II; *unc-119*(ed3) III, oxEx1578
IG1241	*sta-2*(ok1860) V
RB2519	*drh-1*(ok3495) IV
SX2375	*drh-1*(WT)(mjIs225) in JU1580
SX2862	*sta-1*(ok587) IV ; *rde-1*(ne219) V
SX2914	*sta-1*(ok587) IV
SX2917	*sta-1*(ok587) IV ; *sta-2*(ok1860) V
SX2966	mjSi88 II ; *unc-119*(ed3) III ; *sta-1*(ok587) IV
SX3020	*rde-1*(ne219) V ; *sid-3*(ok973) X
SX3024	*sid-3*(ok973) X
SX3034	*sid-3*(tm342) X
SX3035	*sid-5*(tm4328) X
SX3037	mjSi88 II ; *unc-119*(ed3) III ; *sta-1*(ok587) IV ; *sid-3*(ok973) X
SX3046	*sid-1*(qt129) V
SX3072	*sid-2*(qt142) III
SX3112	*sid-2*(qt42) III
WM27	*rde-1*(ne219) V
